# Improving Engagement Among Patients With Chronic Cardiometabolic Conditions Using mHealth: Critical Review of Reviews

**DOI:** 10.2196/15446

**Published:** 2020-04-08

**Authors:** Kamila Cheikh-Moussa, Jose Joaquin Mira, Domingo Orozco-Beltran

**Affiliations:** 1 University Hospital San Juan de Alicante The Foundation for the Promotion of Health and Biomedical Research San Juan de Alicante, Alicante Spain; 2 Health Psychology Departament Miguel Hernández University Elche Spain; 3 Clinical Medicine Department Miguel Hernández University San Juan de Alicante, Alicante Spain

**Keywords:** mHealth, patients, telemedicine, engagement, chronic disease, cardiovascular disease, diabetes, obesity

## Abstract

**Background:**

The burden imposed by cardiometabolic diseases remains a principal health care system concern. Integration of mobile health (mHealth) interventions is helpful for telemonitoring of these patients, which enables patients to be more active and take part in their treatment, while being more conscious and gaining more control over the outcomes. However, little is known about the degree to which users engage, and the extent to which this interaction matches the usage pattern for which mHealth interventions were designed.

**Objective:**

The aim of this study was to describe the characteristics and results of studies on mHealth solutions that measured the effects of interventions with patient engagement in the context of chronic cardiometabolic diseases.

**Methods:**

A critical review of systematic reviews was conducted to recover data on interventions focused on the engagement of patients with chronic cardiometabolic diseases using mHealth technologies. Articles (from January 1, 2010) were searched in the Medlars Online International Literature Medline (Medline/Pubmed), Embase, Cochrane Library, PsycINFO, and Scielo databases. Only studies that quantified a measure of engagement by patients with cardiometabolic disease were included for analysis. The Critical Appraisal Skills Programme (CASP) was used to determine included studies considering the quality of the data provided. The Scottish Intercollegiate Guidelines Network (SIGN) checklist was used to assess the quality of the evidence according to the methodology used in the studies reviewed. Engagement was defined as the level of patient implication or participation in self-care interventions. Engagement measures included number of logs to the website or platform, frequency of usage, number of messages exchanged, and number of tasks completed.

**Results:**

Initially, 638 papers were retrieved after applying the inclusion and exclusion criteria. Finally, only three systematic reviews measuring engagement were included in the analysis. No reviews applying a meta-analysis approach were found. The three review articles described the results of 10 clinical trials and feasibility studies that quantified engagement and met the inclusion criteria assessed through CASP. The sample size varied between 6 and 270 individuals, who were predominantly men. Cardiac disease was the principal target in the comparison of traditional and mHealth interventions for engagement improvement. The level of patient engagement with mHealth technologies varied between 50% and 97%, and technologies incorporating smartphones with a reminder function resulted in the highest level of engagement.

**Conclusions:**

mHealth interventions are an effective solution for improving engagement of patients with chronic cardiometabolic diseases. However, there is a need for advanced analysis and higher-quality studies focused on long-term engagement with specific interventions. The use of smartphones with a single app that includes a reminder function appears to result in better improvement in active participation, leading to higher engagement among patients with cardiometabolic diseases.

## Introduction

### Background

Historically, patient engagement has been an essential factor to obtain better results and outcomes in health care interventions. A medical or health care service focuses on active participation of the patient during treatment to improve the outcome and enhance the patient’s health [1-4]. Such interventions further increase the patient’s awareness of taking more control over their health status, whereas traditional health care usually places the patient in a passive role during treatment. Recent studies have shown that higher patient engagement levels can be achieved using novel technological solutions such as mobile apps and e-devices [5-10]. Patient engagement is particularly relevant in cases of chronic diseases in which the outcome of the intervention largely depends on lifestyle choices and self-care capacity, along with the manner in which that patients cope with their daily lives.

### Mobile Health in Chronic Cardiometabolic Diseases

Mobile health (mHealth) technologies such as the use of mobile phones and other wireless technologies in medical care can empower patients, with promising possibilities for optimizing health systems, enhancing health and care outcomes, and reducing resource consumption [11]. The use of mHealth technologies is starting to become more widespread in the case of cardiometabolic diseases (diabetes, coronary diseases, and obesity), which remain top priorities and principal concerns of all health care systems [12-14]. Indeed, recent studies have shown better clinical indicators, more healthy behaviors, and greater use of preventive behaviors among patients that are motivated by participation in their own health care [15,16].

The integration of virtual telemonitoring interventions [3,4] meets the challenge of ensuring the adequate use of health services by controlling expenditures on medication and diagnostic tests, and in the delivery of effective monitoring of patients at the same time, offering patients greater control of their conditions. These telemonitoring technologies include medical and public health practices supported by mobile devices such as mobile phones, patient monitoring devices, personal digital assistants, and other wireless devices [2]. These devices permit patients to make informed decisions on their care options and to have direct interaction with health providers. In addition, health providers can have exhaustive control of the symptoms, adherence to treatment, and engagement of the patients with treatment indications [6-8].

### Patient Engagement

Although recent reviews have shown that mHealth technologies are both effective and growing in popularity [7,9], their effectiveness heavily relies on patient engagement, as a lack of such engagement can result in treatment failure. Patient engagement refers to “the process of building the capacity of patients, families, carriers, as well as health care providers, to ease and support the active participation of patients in their care, to enhance safety, quality and people-centeredness of health care service delivery” [17]. Despite the many definitions of patient engagement, they all share an underlying theme: the facilitation and strengthening of the role of those using services as coproducers of health, and health care policy and practice. Overall, patient engagement involves active partnership at various levels across the health care system, including direct care, organizational design and governance, and policy making, to improve health and health care.

Accordingly, the factors that may positively influence this relationship include increases in the patients’ perceived benefit of interacting with the agent (by providing useful information or entertainment) [5], decreases in their perceived costs [6], increases in their perceived investment in the system, and decreases in their perceptions of viable alternatives to using the system [7]. These factors all tend to increase user commitment in continuing to participate with the agent and thereby ensures their long-term engagement [3,18,19].

Engagement has been related to the coproduction concept, which describes how patients may individually or collectively engage in the delivery of their treatments and services in partnership with doctors and other health professionals [20]. Thereby, the engagement concept considers the inclusion of patients and family members as active members of the health care team and collaborative partnerships with providers and provider organizations.

The World Health Organization declared engagement as a main factor indicating patient safety in health care, which is considered a global challenge. However, the majority of studies and policies are focused on issues related to hospital care. Therefore, such policies also need to be adapted for primary care because most health care is now offered in this setting. Recently, mHealth interventions have been proposed as a solution to improve patient use of health services, thereby increasing their participation in their own care and the safety of treatments. Studies of the Valcrònic research group demonstrated higher engagement and safer usage of treatments, along with better knowledge of the disease for patients using daily mHealth devices [8-10,18-25]. However, little is known about the degree to which patients engage and if this matches the usage pattern of mHealth interventions.

Based on this background, the engagement concept has been addressed from different viewpoints, and thus it is important to clarify its primary purpose in the management of health results. Several review articles on the topic have led to renewed interest in the concept of engagement. Therefore, from a practical point of view, a comprehensive review or meta-analysis could offer more information about engagement usage in patients with cardiometabolic diseases considering their prevalence, high consumption of health care resources, and recent findings showing that a high level of patient engagement was related to better health care outcomes [16,26].

Toward this end, the aim of this study was to describe the characteristics and results of mHealth solutions based on systematic reviews and meta-analyses of studies that investigated and measured the effects of interventions according to patient engagement in the context of chronic cardiometabolic diseases.

## Methods

### Study Design

We conducted a critical review of systematic reviews and meta-analyses on studies of mHealth interventions focused on the engagement of patients with cardiometabolic diseases. The Preferred Reporting Items for Systematic Reviews and Meta-analyses (PRISMA) protocol was applied [27].

### Data Sources

#### Search Strategy

Studies were searched from the Medlars Online International Literature (Medline) database, via PubMed, Embase, The Cochrane Library, American Psychology Association (PsycINFO), and Scientific Electronic Library Online (Scielo) databases, using Medical Subject Headings (MeSH) and key terms searched in the title and abstracts using the Boolean connectors in Textbox 1.

**Boolean connectors used for database search.**

(“TREATMENT ADHERENCE AND COMPLIANCE” [title/abstract] OR “HEALTH BEHAVIOR” [title/abstract] OR “ENGAGEMENT” [title/abstract]) AND (“TELEMEDICINE” [title/abstract] OR “TELECARE” [title/abstract] OR “telehealth” [title/abstract] OR “homecare” [title/abstract] OR “telemonitoring” [title/abstract] OR “home monitoring” [title/abstract] OR “remote monitoring” [title/abstract] OR “ehealth” [title/abstract] OR “telerehabilitation” [title/abstract] OR “mobile health” [title/abstract] OR “mhealth” [title/abstract] OR “assisted living” [title/abstract] OR “technology-based” [title/abstract] OR “information technology” OR “health communication” [title/abstract] OR “internet-based” [title/abstract] OR “web-based” [title/abstract] OR “on-line” [title/abstract] OR “smartphones” [title/abstract] OR “mobile apps” [title/abstract] OR “mobile phone” OR “monitoring devices” [title/abstract])

The filters “Humans”, “Meta-Analysis”, “Review”, and “10 years” were used to identify all relevant, peer-reviewed systematic reviews and meta-analyses of interventional studies published as of January 1, 2010.

The studies included among the retrieved reviews were also checked for compliance with inclusion criteria using the Critical Appraisal Skills Programme (CASP). A manual search of the references was performed to reduce possible publication bias and to identify undetected studies. Both systematic reviews and meta-analyses were considered regardless of the country, institution, author, or language.

#### Inclusion and Exclusion Criteria

Articles were eligible for inclusion in the analysis when they met the following criteria: systematic review or meta-analysis of randomized or controlled clinical trials, or a feasibility, usability, and utility (FUU) design measuring the effects of mHealth interventions on engagement of adult (>18 years old) patients with chronic cardiometabolic conditions. Studies were excluded if they assessed nonchronic diseases, cancer, respiratory disease, mental health, substance abuse, did not involve engagement measurement or telemonitoring, or assessed a pediatric population.

#### Type of Intervention

Reviews or meta-analyses that investigated the effectiveness of interventions applying text messages, smartphones or phones with internet, mobile apps, videos, and websites were all considered.

#### Outcome Measures

The main outcome assessed was patient engagement, which was measured as the number of logs to the website or platform, frequency of usage, amount of messages exchanged, and task completion.

### Information Extraction

Quantitative and qualitative information on patient engagement suffering from chronic cardiometabolic conditions was extracted from systematic reviews or meta-analyses. An electronic form was developed to group the papers by the following items: review, author information, year, study design, participants, intervention, outcomes assessed and comparisons performed, pooled sizes of outcomes meta-analyzed, and the main conclusion. The availability of meta-analysis studies and the quality of evidence provided by the studies included in the systematic reviews were considered in the selection of studies for subsequent review. From this initial selection, a reconceptualization of the findings provided by systematic review studies was conducted.

Two authors assessed the relevance and adequacy of the studies (CM and JM). The selection was valid when the concordance between the two authors (kappa index) was higher than 0.80 (representing a high or very high strength of concordance). The third author was available for arbitration in case of persistent disagreements, followed by consensus among all authors.

### Quality Assessment

The CASP tool was used for appraisement by two authors. Reviews that did not meet at least 6 of 10 screening items were excluded. The Scottish Intercollegiate Guidelines Network (SIGN) criteria were applied to classify the quality of the evidence provided by the retrieved reviews.

## Results

### Retrieved Papers

**Figure 1:**
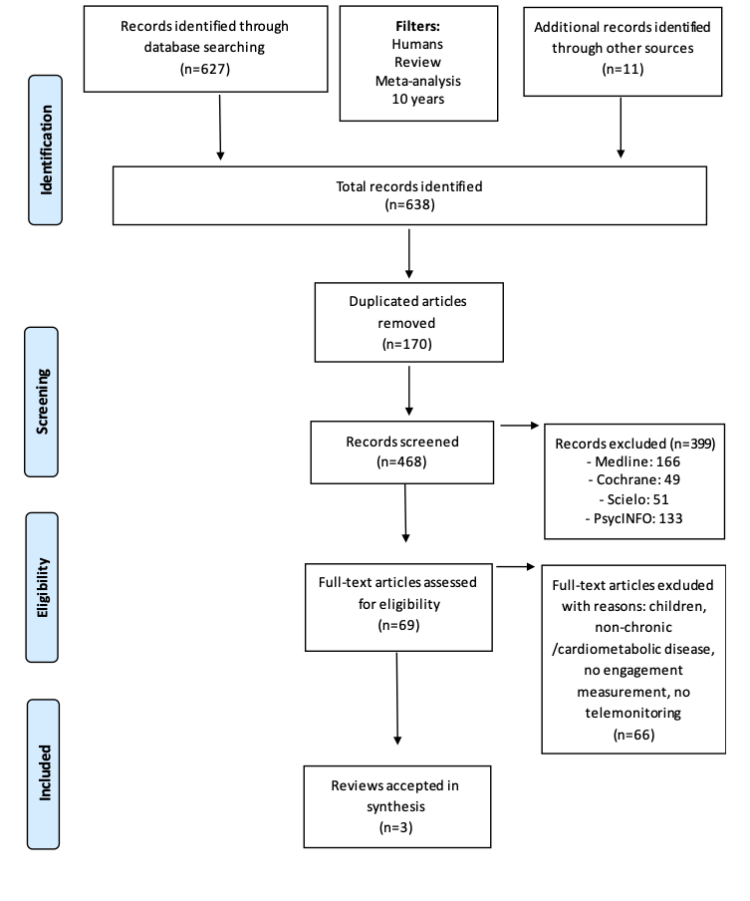
Flowchart of article selection process.

The initial search returned 627 records (186 Medline, 50 Cochrane, 51 Scielo, 170 PsycINFO, and 170 Embase); no meta-analysis studies were detected. Eleven additional articles were identified from the reference lists of the studies included in the initial screen. After applying the inclusion and exclusion criteria and removal of duplicates, three systematic reviews with engagement evaluation in cardiometabolic patients that met all quality criteria were extracted and included in the final analysis [28-30]. Figure 1 summarizes the overall search strategy and article selection process.

The three reviews ultimately included for analysis only described the main results without providing quantitative data and with no statistical pooling analysis performed. These reviews included evidence from a total of 150 papers (93 clinical trials and 57 FUU or qualitative studies), but only 10 of these studies quantified engagement specifically. Therefore, the results of these 10 studies were extracted from the reviews and their findings were also summarized. The remaining 140 empirical studies focused on the fulfillment of tasks, rather than engagement, and did not include specific measurements of engagement.

### Concordance Between Reviewers

The concordance (kappa index) between the authors identifying and extracting information from these studies was 90%, demonstrating very good agreement to reach consensus about the inclusion of articles without requiring intervention of a third author. The assessment of quality was performed using the CASP tool and returned a score of 8-10 for the accepted systematic reviews. All three of the selected systematic reviews studies were published within the last four years (2016-2018).

### Study Characteristics

Table 1 summarizes the measures and interventions used in the empirical studies included in the systematic reviews analyzed. Cardiac disease was the main chronic disease of focus [28], followed by diabetes mellitus type 2 [29,30] and obesity [29]. These studies described the characteristics of different engagement tools, collected information of mHealth interventions that examined the usage of smartphones or computer self-assessment on health outcomes [28,29], and described how text messages were used as reminders to improve engagement in patients with type 2 diabetes [30].

**Table 1:** Characteristics of systematic reviews and patient engagement.

Review	Quality/source	Disease	mHealth type/source	Engagement measure (%)	Description
Hamilton et al [28]	SIGN^a^: ++ N^b^: 7 of 9 studies 2 RCT^c^ 2 CT^d^ 3 FUU^e^	Cardiac disease and heart failure	Smartphone	Outdoor walking-based exercise program Follow-up > 180 days Adherence to protocol Engagement with technology Adherence Visits (0 to 6 weeks or 4 months) Response to blood glucose reminder measure Access to core sessions Access to optional sessions	Step counter Blood pressure self-measurement and data entry Daily readings Lifestyle counseling and usual care Daily messages and tasks Educational material and videos Medication reminders Physical activity prompts Screenings and surveys Wellness diary Relaxation audio files Community care team Internet Web portal for viewing of patient data Text messages Video and telephone mentoring
Perski et al [29]	SIGN: + N: 2 of 117 studies 1 RCT 1 FUU	Diabetes Obesity	Computer-assisted self-management program with or without expert support	Visited (0 to 6 weeks) Visited (6 weeks to 4 months) Access to core sessions Access to optional sessions	Internet-based website “My Path” (“*Mi Camino*”) for self-management with or without the addition of social support from the health care team and peer group meetings. 12 weekly online sessions to control and review weight goals.
Nelson et al [30]	SIGN: + N: 1 of 24 studies 1 FUU	Type 2 diabetes	Text message reminders for blood glucose measurement	Response to blood glucose reminder measure	Three text messages per week requesting blood glucose readings and three text messages with appointment reminders before each scheduled appointment.

^a^SIGN: (-) low, (+) acceptable, (++) high quality.

^b^N: number of eligible studies.

^c^RCT: randomized controlled clinical trial.

^d^CT: controlled clinical trial.

^e^FUU: feasibility, usability, and utility.

There was substantial variation in how intervention engagement was reported across the three studies [28-30]. The FUU-based studies of the mHealth interventions delivered for cardiovascular diseases are typically analyzed based on significant improvement in the quality of life with higher rates of patient engagement for patients unable to attend traditional center-based programs [28]. Engagement with mHealth interventions rates decreased over time, and longer interventions, patients of older age, more familiarity with the use of these technologies, and lower health literacy showed progressively poor engagement [29,30]. The impact of these mHealth interventions to reduce hospital utilization was inconsistent [29].

Since all three reviews were descriptive in nature, no quantitative results were discussed, and there was no statistical pooling. These studies included only four randomized trials [31-34], one controlled trial [35], and five FUU designs [36-40] in which engagement outcomes were quantified. One of the systematic reviews [28] included six empirical studies that assessed engagement with mHealth strategies in which outcomes were compared with those in patients receiving traditional care [31,32,34,35,37,38].

### Participants and Study Design

The sample size of studies included in the reviews varied between 6 and 270 individuals [33,36]. The age of the patients was between 50 and 66 years [31,37]. Men were in higher proportion in general, although one of the studies included equal numbers of men and women [33]. Most of the studies included two groups for assessment: the intervention group (mHealth, S) and the traditional control group (TC); only one study included a three-group design (Tables 2 and 3). The follow-up period varied from 3 months to 4 months. The studies used different measures of engagement, and the global percentage of adherence/engagement or daily readings was used.

**Table 2:** Characteristics and design quality of included reviews.

Review and included studies		Participants, n (% male)	S^a^ (% male); TC^b^, (% male)	Age (years); median (IQR), mean (SD), or range		Design	Evidence quality (SIGN^c^)	
**Hamilton et al [28]**								
	Scherr D, 2006 [37]	20	S1, 14 (93); S2, 6 (83)	50 (14)		FUU^d^	+	
	Scherr D, 2009 [31]	120	S, 66 (69.5); TC, 54 (72)	66 (64-74)		RCT^e^	++	
	Worringham C, 2011 [36]	6	—^f^	53.6 (42-67)		FUU	+	
	Blasco A, 2012 [32]	203 (80)	S, 102 (81); TC, 101 (79)	60.6 (23.8)		RCT	++	
	Seto E, 2012 [35]	100	S, 50 (82); TC, 50 (76)	53.5 (14)		CT^g^	+	
	Varnfield M, 2014 [34]	94	S, 53 (91); TC, 41 (83)	52.13 (9.2)		RCT	+	
	Forman D, 2014 [38]	26 (77)	—	59 (43-76); 33%>65		FUU	+	
**Nelson et al [30]**								
	Fischer H, 2012 [39]	47	—	50-59		FUU	+	
**Perski et al [29]**								
	Glasgow R, 2011 [33]	270	S1, 137 (54.7); S2, 133 (48.9)	57.8 (9.3)	RCT			++
	Arden-Close E, 2015 [40]	132	S1, 137 (54.7); S2, 133 (48.9)	57.8 (9.3)	FUU			+

^a^S: mHealth study group.

^b^TC: traditional control group.

^c^SIGN: (-) low, (+) acceptable, (++) high quality.

^d^FUU: feasibility, usability, utility.

^e^RCT: randomized clinical trial.

^f^Not available.

^g^CT: controlled clinical trial.

**Table 3:** Outcomes of engagement using mHealth interventions.

Review and included studies		Disease	Study period (months)	mHealth type	Non-mHealth	Result
**Hamilton et al [28]**						
	Scherr D, 2006 [37]	S^a^1: CHF^b^; S2: HT^c^	3	Clinical app	TC^d^ Blood pressure automatic monitor	S1: 94%; S2: 84%; follow-up >90 days
	Scherr D, 2009 [31]	S/TC: CHF	6	Clinical app, email	TC	S: 95% adherence
	Worringham C, 2011 [36]	S: ACS^e^ TC: none	1.5	Real-time monitoring post exercise sessions, emergency mobile phone contact	none	87% of sessions (outdoor walking-based exercise program) completed
	Blasco A, 2012 [32]	S/TC: CVD^f^ and risk factors^g^	12	Wireless app protocol, web portal	TC	98% completed >50% of sessions; 83% completed >75% of sessions
	Seto E, 2012 [35]	S: CHF	6	Clinical app, email, text messages, website	TC Telephone contact	S: 50% adherence in 80% of patients; 80% adherence in 66% of patients; 95% adherence in 32% of patients; follow-up >180 days
	Varnfield M, 2014 [34]	S/TC: myocardial infarction	6	Smartphone	TC Qualitative patient and clinician feedback	S: 94% adherence; TC: 68% adherence (*P*<.05)
	Forman D, 2014 [38]	S: CHF TC: none	1	Heart coach app	TC Qualitative patient and clinician feedback	90% daily engagement with technology
**Nelson et al [30]**						
	Fischer H, 2012 [39]	S: diabetes II	3	Text message reminder for blood glucose measurements	None	S: 79% of users responded regularly to >50% of blood glucose reminder message prompts
**Perski et al [29]**						
	Glasgow R, 2011 [33]	S1: diabetes II; S2: diabetes II	6	S1/S2: computer-assisted self-management program with human support	None	S1: 66% visits 0-6 weeks, 74% visits 6 weeks to 4 months (*P*=.14); S2: 44% visits 0-6 weeks, 51% visits 6 weeks to 4 months (*P*=.22)
	Arden-Close E, 2015 [40]	S: BMI >30 with HT or diabetes	6-12	Web weight management intervention	None	47% access to core sessions; 47% access to optional sessions; 3% no access

^a^S: mHealth study group.

^b^CHF: congestive heart failure.

^c^HT: hypertension.

^d^TC: traditional control group.

^e^ACS: acute coronary syndrome.

^f^CVD: cardiovascular disease.

^g^Risk factors include tobacco smoking, low-density lipoprotein cholesterol≤100 mg/dL (2.6 mmol/L), hypertension, or diabetes mellitus.

### Mobile Health Technologies

Internet-based technologies were predominant within studies, including the use of smartphones with health-integrated applications [21,28,32,34-37], audio-visual devices (iPod and iPod touch) [28,38], and computers [29,30,39,40] focused on patient self-management. The features of the apps and programs are described in Table 1.

The randomized clinical trial included in the review by Perski et al [29] assessed improvement of engagement with or without instructions provided for use of the mHealth tool (computer) [33], whereas Nelson et al [30] reported the results of a feasibility study in patients with type 2 diabetes who were reinforced with reminder tools such as text messages or email to regulate the measurement of glucose blood levels [39]. Hamilton et al [28] included studies assessing a principal mHealth tool that was combined with other features such as email reminders or Web portals [31,32,35-38]. However, we categorized the study types based on the main technology used in such cases.

When the mHealth intervention was compared with a traditional clinical practice [31,32,34,35,37,38], this was based on face-to-face interventions during regular consultations and diagnosis evaluating equipment, with qualitative information on patients and clinical feedback, as described in Tables 2 and 3.

### Measurement of Engagement

Studies included in the Perski et al [29] review registered the number of visits to a Web-based program or platform [34,40]. Papers extracted from Hamilton et al [28] registered task completion, whereas those included in the Nelson et al [30] review measured the interaction level with agent messages. All empirical studies included in the reviews used self-management systems; however, two of them also included support features as reminders along with a human support system [39,40]. The engagement was measured by the frequency of responses, and two studies considered the number of follow-up days [35,37].

### Findings of the Studies

Overall, mHealth interventions demonstrated high engagement, with the level of patient participation varying between 50% and 97%. Clinical trials from the Hamilton et al [28] review [21,24,35] showed the highest level of engagement (95%) when the interventions were limited to one or two tools using smartphones, whereas a higher number of tools resulted in lower patient interaction. Websites and surveys resulted in less engagement with the intervention (47%-74%) [32,39,40].

### Quality of Evidence

The SIGN scale determined that the three selected systematic reviews met 10 of the 12 items of evidence quality, and the 10 selected studies included in the identified reviews presented a high evidence quality level (Tables 2 and 3).

## Discussion

### Principal Findings

Emerging evidence suggests that mHealth promotes more engagement than traditional interventions. However, few reviews were identified that included a measure of quantitative engagement, and there was poor quality evidence owing to the lack of advanced study designs such as meta-analysis. For the included studies, the duration was not sufficiently long to compensate for the attraction potential that the innovation of these technological solutions implies. In addition, a long-term study (24 months or longer) could decrease participation. Despite the important role that participants should have in mHealth studies, few studies used a specific intervention or measurement of their engagement.

The engagement approach differed among studies, with most focused on the promotion of quality of life and patient behavior with the internet-based intervention [28,31,38]. The feasibility of these interventions was mainly examined as a solution to overcome barriers in health systems in attracting the attention to patients that are compromised by the current high costs [28,34-36]. Analysis of the causes of early dropouts has not been carried out due to incompatibility with these technological solutions (eg, in the case of patients that are technologically illiterate). Consequently, the reviews could not discuss potential solutions for enhancing the usability of mHealth interventions.

A high number of interventions focused on evaluating engagement in patients with cardiac diseases, suggesting that there is a need to approach cardiovascular disease as the leading cause of global mortality (85% due to heart attack and stroke) [41]. High heterogeneity was found for almost all variables, including sample size and sex, and only one study included proportional representation of men and women [29].

### Quality of Evidence

Although the overall internal quality of the selected papers was high, a large number of papers were discarded owing to the low quality of current evidence. The clinical trials retrieved in the detected reviews indicated improvement of engagement with the use of these new strategies. In addition, the FUU studies demonstrated high participation of patients using mHealth interventions; this was reinforced by the high quality of evidence detected with the SIGN scale.

No long-term follow-up period appeared to jeopardize the aim of interventions. This is related to the extensive use of pilot projects in the mHealth sector, which may be due to limited government ownership, multiple barriers around prioritizing funding, overall cost, and acceptance by health authorities and populations [42].

### Reliability and Applicability of the Evidence

There were also differences between the type of mHealth interventions, with higher engagement observed with the use of smartphones. This finding is in line with the results of a survey of the Global Observatory for eHealth in which 62% of the participating countries used mHealth tools for patient monitoring, 69% of which used mobile devices (text, voice, or multimedia reminders) [42].

The characteristics of the engagement assessment tools varied, with some focused on self-measurement and others using tasks or survey complements, with lack of support from physicians or experienced agents as a common feature. These differences reflect the complexity of engagement measurement, which depends of two distinguishable components: the directional component determined by the hedonic quality of the object, and other sources of force intensity (eg, opposition to interfering forces, regulatory fit, overcoming personal resistance) that are included in the construct labeled as “strength of engagement” [43].

Overall, we found that the use of mobile devices was related to an improvement of engagement to mHealth interventions, with a higher level of user commitment observed when a single tool was used with limited functions and with expert support. There is a need for studies with homogeneity in design, engagement measurement, and the type of mHealth tool. Further clinical trials can provide more information about the effectivity and efficiency of mobile technologies in patients with chronic conditions. This could permit comprehensive investigations of outcomes using meta-analysis techniques.

### Limitations

The few retrieved reviews is one of the principal limitations of this analysis, which was due to the lack of studies with engagement measurement. More homogeneity in the design of studies and participant characteristics could facilitate advanced statistical analyses such as systematic reviews and meta-analyses. In addition, these studies could help in the development of new tools focused on patient participation. Moreover, studies on patient social networks could provide more information on engagement.

### Future Research

It is expected that active engagement has a direct impact on the reduction of health costs, but this aspect has rarely been analyzed to date. The number of dropouts or poor engagement with mHealth interventions requires more attention in further studies. Moreover, it could be of interest to study the characteristics of patients who have taken better advantage of these tools. There is also a need to detect patients that obtained relatively less benefits using mHealth interventions, which could help to better personalize the technologies or increase availability for this population.

### Conclusions

Smartphones and other mobile devices with a single tool that includes reminders can improve participation and induce higher engagement with mHealth interventions in patients with chronic conditions.

## Abbreviations

CASP: Critical Appraisal Skills Programme
FUU: feasibility, usability and utility
MeSH: Medical Subject Headings
Medline: Medlars Online International Literature
mHealth: mobile health
PRISMA: Preferred Reporting Items for Systematic Reviews and Meta-Analyses
Scielo: Scientific Electronic Library Online
SIGN: Scottish Intercollegiate Guidelines Network
S: mHealth study group
TC: traditional control group
